# Cognitive training gain transfer in cognitively healthy aging: per protocol results of the German AgeGain study

**DOI:** 10.3389/fnagi.2025.1587395

**Published:** 2025-08-26

**Authors:** Florian U. Fischer, Bianca Kollmann, Dominik Wolf, Alexandra Sebastian, Kristel Knaepen, David Riedel, Andreas Mierau, Nicolas Ruffini, Kristina Endres, Jennifer Winter, Heiko K. Strüder, Gerard N. Bischof, Sofia Faraza, Bernhard Baier, Harald Binder, Alexander Drzezga, Stefan Teipel, Andreas Fellgiebel, Oliver Tüscher

**Affiliations:** ^1^Department of Psychiatry, Psychotherapy and Psychosomatic Medicine, University Medical Center Halle, Halle (Saale), Germany; ^2^Department of Psychiatry and Psychotherapy, University Medical Center Mainz, Mainz, Germany; ^3^Department of Neuropsychology and Psychological Resilience Research, Central Institute of Mental Health (ZI), Mannheim, Germany; ^4^Leibniz Institute for Resilience Research, Mainz, Germany; ^5^Institute of Movement and Neurosciences, German Sport University Cologne, Cologne, Germany; ^6^Department of Sport, LUNEX, Differdange, Luxembourg; ^7^Faculty of Computer Sciences and Microsystems Technology, Kaiserslautern University of Applied Sciences, Zweibrücken, Germany; ^8^Institute for Human Genetics, University Medical Center of the Johannes Gutenberg University Mainz, Mainz, Germany; ^9^Department of Nuclear Medicine, Faculty of Medicine and University Hospital Cologne, University of Cologne, Cologne, Germany; ^10^Institute of Neuroscience and Medicine (INM-2), Molecular Organization of the Brain, Forschungszentrum Jülich, Jülich, Germany; ^11^Department of Psychosomatic Medicine and Psychotherapy, University Medical Center Rostock, Rostock, Germany; ^12^German Center for Neurodegenerative Diseases (DZNE), Rostock/Greifswald, Germany; ^13^Department of Neurology, University Medical Center of the Johannes Gutenberg University Mainz, Mainz, Germany; ^14^Institute of Medical Biometry and Statistics (IMBI), University Medical Center Freiburg, Freiburg, Germany; ^15^German Center for Neurodegenerative Diseases (DZNE), Bonn-Cologne, Germany; ^16^Clinic for Psychiatry, Psychosomatics and Psychotherapy, AGAPLESION Elisabethenstift, Darmstadt, Germany; ^17^German Center for Mental Health (DZPG), Site Halle-Jena-Magdeburg, Halle (Saale), Germany; ^18^Institute of Molecular Biology (IMB) Mainz gGmbH, Mainz, Germany

**Keywords:** cognitive training, cognitive transfer, healthy aging, functional connectivity, interhemispheric structural connectivity

## Abstract

**Introduction:**

Cognitive decline is part of the normal aging process, but also a major risk factor for dementia. Cognitive training interventions aim to attenuate cognitive decline, but training gains need to be transferable to untrained cognitive abilities to influence everyday function. Furthermore, the neurobiological basis of cognitive training gain transfer remains elusive. A possible candidate is increased bilateral hemisphere usage enabled by efficient structural connectivity, especially of prefrontal regions. Therefore, the present multicentric study used a cognitive training intervention to demonstrate training transfer and identify neurobiological modulators of successful transfer.

**Methods:**

In total 235 subjects were enrolled in AgeGain; 180 underwent a broad 4-week cognitive training intervention at three study sites. Pre- and post-training neuropsychological testing was conducted and successful transferers were identified according to preregistered definitions. Pre-training, subjects underwent diffusion and functional MRI to assess interhemispheric connectivity, measured as microstructural integrity of the corpus callosum and lateralization of functional activation patterns during a cognitive control task. Logistic regression models were estimated to predict successful transfer based on structural connectivity and bilateralization of activation patterns.

**Results:**

Out of 180 subjects, 74 showed short-term training gain transfer that was maintained over 3 months in 19 subjects. Neither microstructural integrity of the corpus callosum, nor bilateralized activation predicted training gain transfer alone. However, their interaction was associated with long-term transfer over 3 months: subjects with higher mean diffusivity of the corpus callosum and more bilateral functional activity or conversely with lower diffusivity of the corpus callosum and more lateral functional activity were more likely successful long-term transferers.

**Discussion:**

We demonstrated successful training gain transfer in 41.1% of subjects, among whom 25.7% maintained the transfer over 3 months. Successful long-term transfer of training gains may depend on divergent mechanisms of structural and functional connectivity, which may explain previous heterogeneous results in the literature.

**Trial register:**

German Clinical Trials Register (DRKS), ID: DRKS00013077. Registered on November 19th 2017.

## 1 Introduction

Cognitive decline is a common process in normal aging and may deteriorate to mild cognitive impairment (MCI) and dementia (Murman, 2015). Cognitive training has been proposed as a means to attenuate the cognitive decline during normal aging. One of the most common forms of application of cognitive training is computerized training, showing good effectiveness in improving trained task performance, e.g., Guye et al. (2017). Among other forms of computerized training, training in the lab is still widely used because it allows for higher application standardization and better control of possible training biases (i.e., control over dosage of training, ruling out factors biasing attention, timing of training).

Improvements in trained tasks that also lead to improvement in untrained tasks is generally referred to as cognitive transfer. Successful transfer is highly desirable as it could indicate that training gains will have a positive impact on everyday cognitive functioning of subjects. Additionally, transfer effects should be maintained over time if they are to support healthy aging. A vast amount of studies on cognitive training effects have focused on working-memory training with heterogeneous results considering transfer effects (Ripp et al., 2022; Karbach and Verhaeghen, 2014). Another important cognitive domain entails higher-level executive functions, requiring a complex interplay between several executive functions, e.g., inhibition or working memory (Diamond and Wright, 2014). These higher-level executive functions become increasingly important for everyday functioning in older age and might therefore benefit specifically from training (Willis et al., 2006). One of these functions is logical reasoning, for which we found transfer effects in the past (Wolf et al., 2014).

While transfer effects of cognitive training have repeatedly been found, the neurobiological basis of cognitive transfer is not well understood. According to the hemispheric asymmetry reduction of older adults model (HAROLD; Cabeza, 2002), elderly participants might compensate cognitive decline on a neural level by increasing bilateral activation. We were able to show this phenomenon under increased task demands in young participants (Sebastian et al., 2013b) and on lower task demands in healthy elderly participants performing inhibition tasks of different quality (Sebastian et al., 2013a). Similarly, the scaffolding theory of aging and cognition (STAC; Reuter-Lorenz and Park, 2014) proposes that learning and skill acquisition, as occurs during cognitive training, results in the compensatory use of additional brain circuits to enable good task performance. Scaffolding is assumed to occur mainly in prefrontal brain regions (Reuter-Lorenz and Park, 2014), which are also the same regions which support executive functioning (Diamond and Wright, 2014), a key domain assessed in cognitive training. Consequently, bilateral brain activation might be a prerequisite for successful cognitive transfer (Wolf et al., 2018). Good structural integrity of the white matter pathways connecting the hemispheres appears to be a prerequisite in order to achieve bilateral activation patterns in the brain. Indeed we previously demonstrated that long-term transfer of cognitive training gains in healthy elderly were related to the structural integrity of the corpus callosum (Wolf et al., 2014).

The two aims of the present study are as follows: First, to reproduce the successful transfer of gains in a trained cognitive task to an untrained cognitive task as in the precursor study by Wolf et al. (2014). Second, to investigate the structural and functional neurobiological mechanisms underlying successful short- and long-term training transfer in healthy aging in more detail as per the primary objectives of the AgeGain study protocol (Wolf et al., 2018). The AgeGain study is a multicenter randomized controlled cognitive training study in healthy older adults. Assessments include structural integrity of the corpus callosum by means of diffusion tensor imaging (DTI), as well as bilaterality of task-related cortical activity using the functional MRI-derived lateralization index (LI). The LI quantifies brain activation lateralization during functional imaging tasks. In the current study, a response inhibition task, combining several inhibitory processes of differing task demands, has been chosen to elaborate on previous findings (Sebastian et al., 2013a). To control the practive effect on the transfer task, a control group was included that did not receive cognitive training. Based on previous findings from our group and an ex-ante definition of transfer, we expected to observe short- and long-term training gain transfer in a higher-level executive functions task in a subgroup of our participants. We expected long-term training gain transfer to be related to better structural integrity of the corpus callosum. Additionally, we expected higher bilateral processing in successful transferers, as demonstrated by increased bilateral activation during a response inhibition task measured in the scanner. Here, increased bilateral brain activation should be most evident with increasing cognitive task demands.

## 2 Methods

### Design and study sample

The data used in this study was taken from the AgeGain study sample, a multicentric, multimodal imaging, interventional, longitudinal, parallel group study using a RCT design for subject group assignments. The study was preregistered with the German Clinical Trials Register (DRKS), ID: DRKS00013077, on November 19th 2017. The aim of the AgeGain study was on the one hand to reproduce successful transfer of training gains in a cognitive training intervention, and on the other hand to investigate neurobiological modulators of cognitive training gain transfer. To this end, 235 cognitively healthy elderly subjects aged over 59 were recruited at the university medical centers in Mainz and Rostock and the German Sport University Cologne between 2016 and 2019 by means of newspaper announcements and flyers. One hundred and eighty one subjects underwent a 4-week cognitive training intervention as well as pre-training MRI and pre-training, post-training and follow-up neuropsychological examinations (see below). Fifty four subjects were randomly assigned to the control group that did not receive cognitive training or MRI. Written informed consent was obtained by all subjects and the study protocol was approved by the respective local ethics commission. Exclusion criteria were psychological, neurological or cognitive illnesses, cardiovascular disease, disorders restricting physical capacity, diabetes, medication affecting cognitive performance, insufficient German language skills, current participation in other trials and MRI contraindications. The design of the study has been published previously (see Wolf et al., 2018 for details).

Of the 181 subjects in the intervention group that received cognitive training, 180 subjects with complete neuropsychological assessment and cognitive training data were included. Of these, 166 received diffusion-weighted imaging (DWI) scans, while 139 had complete functional MRI (fMRI) scans. For an overview of respective demographic data, please refer to Table 1. In order to assess retest effects of transfer tasks, the 54 subjects that did not receive cognitive training nor MR imaging were included as a control group.

**Table 1:** Descriptive group statistics of transferers vs. non-transferers.

	**STT**	**no STT**	**p**	**LTT**	**no LTT**	**p**
**N**	74	106		19	161	
**Female**	39	51		11	79	
**Age**	68.8 ± 6.0	67.9 ± 5.2	0.4219	70.8 ± 7.2	68.0 ± 5.3	0.1078
**Education**	15.8 ± 2.9	15.8 ± 2.6	0.8236	15.3 ± 2.9	15.9 ± 2.7	0.4551
**HAWIE-R**	116.6 ± 10.9	117.2 ± 11.2	0.9420	116.0 ± 8.8	117.1 ± 11.3	0.5387
**Training task**	459.4 ± 18.6	462.9 ± 21.70	0.0319*	451.6 ± 24.7	462.6 ± 19.7	0.0143^*^
**Transfer task**	23.7 ± 4.2	26.6 ± 3.5	< 0.0001	23.1 ± 3.9	25.6 ± 4.0	0.0188^*^

Mean ± standard deviation. STT, short-term transfer; LTT, long-term transfer; Education, in years; HAWIE-R, revised Hamburg Wechsler Intelligence Test; Training task, logical reasoning training task performance at baseline; Transfer task, Leistungspruefsystem subtest 4 executive function cognitive training gain transfer task performance at baseline; P, *p*-values of Wilcoxon test;

^*^statistically significant at *p* < 0.05 level.

### Neuropsychological examination

The neuropsychological examination was conducted immediately prior to the 4-week cognitive training intervention (pre-training), immediately after the cognitive training intervention (post-training) and as a follow-up examination 3 months after the cognitive training intervention. The examination included several common cognitive measures from multiple domains. The cognitive measure used as an endpoint for the cognitive training gain transfer in this study was the Leistungspruefsystem (achievement measurement system) subtest 4 (LPS4; Horn, 1983), which refers to fluid intelligence and is comparable to the Raven Matrices. In order to assess general intelligence, a short version of the revised Hamburg Wechsler Intelligence test (HAWIE-R) was applied (Tewes, 1991).

### Cognitive training

Cognitive training was conducted as three 60-min sessions per week over the course of four weeks (12 in total). Cognitive training was conducted within two dedicated rooms, where each subject was seated in front of a personal computer equipped with headphones and the necessary training software. Subjects were instructed not to seek advice or contact with other subjects during training and opaque screens where used to shield subjects from each others' view. To rule out any biasing effects from differences in training dose or training administration, the cognitive training was applied under supervision of an experimenter. Further, to rule out mere practice effects on the transfer tasks assessed, a control group was included, that did not receive any training.

The cognitive training intervention consisted of computerized cognitive training tasks spanning several cognitive domains. Specifically, executive functions, memory and information processing speed were trained using the subtests “comparisons,” “searching,” “logic,” “anagrams,” and “complete a logical block” within the computer program Cogpack (Marker, 2008). Attention capacities were trained using the subtests “alertness” and “divided attention” within the computer program TAP (Zimmermann and Fimm, 2015). For the training of working memory, the subtests “complex span” and “tower of fame” within the computer program TATOOL (Von Bastian et al., 2013) were employed. Subjects could familiarize themselves with the training tests in one test session.

### Cognitive training transfer

The cognitive training gain transfer was defined ex-ante and previously published along with the study protocol (Wolf et al., 2018). Cognitive training gain transfer was divided into a short-term and a long-term component, both defined as dichotomous variables. For short-term transfer, subjects needed to fulfill the following conditions: (1) an improvement in the logical reasoning training task in the last training session compared to the second training session, (2) an improvement in the untrained fluid intelligence task (LPS 4) from the pre-training neuropsychological examination to the post-training neuropsychological examination, (3) the improvement in the untrained intelligence task needed to be greater than the mean difference between examinations in the control group that did not undergo cognitive training, in order to account for retest effects.

For long-term transfer, subjects needed to fulfill the conditions for short-term transfer as well as two additional conditions: (1) maintenance of the improvement in the untrained fluid intelligence task from post-training to follow-up examination, (2) the difference from post-training to follow-up needed to be greater than the group mean difference between examinations in the control group, in order to account for retest effects.

### Hybrid response inhibition task

A so-called hybrid response inhibition (HRI) task was applied, combining characteristics of a Simon, Go/NoGo, and Stop signal task. It enables the identification of component-specific neural task activation for response interference inhibition, action withholding, and action cancellation, respectively. Subjects underwent three runs of the task, preceded by a brief practice trial outside of the scanner on a laptop to ensure that all participants understood the task. The task was programmed in Presentation (version 13.0, http://www.neurobs.com). Participants responded via a button press with their left or right index finger, using a LUMItouch Box, which was placed in the left and right hand. Four different conditions were presented: a congruent go condition (62.5% of trials), incongruent go condition (12.5%), NoGo condition (12.5%), and a stop condition (12.5%). Notably, the stop and NoGo conditions entailed only congruent target stimuli. The stop signal delay (SSD) in the stop condition was adaptive to participant's performance, using a staircase procedure, to ensure a rate of about 50% of successful inhibitions per participant per run. More precisely, following a correct response, the response window was increased by 50 ms on the subsequent run, while it was decreased by 50 ms after unsuccessful stop trials, where a commission error occurred. The initial SSD of each run was set to 220 ms. Each run consisted of 160 trials in pseudo-randomized order (for more details, see Sebastian et al., 2013b).

Mean reaction times and accuracies were computed from the Presentation output using Matlab 2012b (The Mathworks Inc, Natick, Massachusetts, USA). The interference effect was calculated through the subtraction of the mean reaction times (RT) of congruent go trials from that of the incongruent go trials. The stop signal reaction time (SSRT) was estimated using the integration method. Here, go RTs of correctly answered go trials (congruent and incongruent ones combined) are ranked according to response speed. Of note, omissions (i.e., missing responses on go trials) are replaced with the respective maximum go RT for congruent and incongruent trials over all runs, respectively (Verbruggen et al., 2019). Subsequently, the probability of responding given a stop signal is calculated, including premature responses on unsuccessful stops and mean SSD (Verbruggen et al., 2019). The stop process finishes at the n-th RT, which is the amount of RT in the distribution of go trial RTs times the likelihood of responding given a stop signal. The mean SSD is then subtracted from the n-th percentile of the ranked go RTs to obtain the SSRT (Verbruggen and Logan, 2009). Based on the recommendations by Verbruggen et al. (2019), *n* = 6 participants with a probability < 0.25 of responding given a stop signal were excluded. Further, participants with too many go omissions (i.e., no response on go trials) were excluded. For this, the cutoff was set to >15% go omissions, resulting in *n* = 3 participants, that had to be excluded from further analyses.

### Structural imaging data and processing

T1 and DWI images were acquired on three different Siemens 3T scanners - a Prisma in Cologne, a Trio in Mainz and a Verio in Rostock. The same T1 and DWI sequences were implemented on these scanners. Specifically, for T1 weighted images a Generalized Autocalibrating Partial Parallel Acquisition sequence was used with a repetition time of 1,900 ms, an echo time of 2.52 ms, an isotropic voxel size of 1mm ^3^. For DWI images, an echo planar multiband sequence was used with a multiband factor of 3, a repetition time of 5,500ms, an echo time of 104ms and an isotropic voxel size of 2*2*2mm ^3^. 64 diffusion gradient directions were sampled at *b* = 2,000s/mm ^2^. Additionally, two *b* = 0 images were acquired as well as one *b* = 0 image with inverted phase encoding direction.

T1 weighted data were tissue segmented and non-linearly registered to standard MNI space using DARTEL included in SPM12 (Ashburner et al., 2014). Spatial distortions in the diffusion-weighted images were corrected using Eddy from the software package FSL 6.0.4 (Andersson and Sotiropoulos, 2016). Diffusion tensors were fitted to the diffusion-weighted images using an iterated weighted least squares approach, and fractional anisotropy (FA) and mean diffusivity (MD) were calculated from the tensors' eigensystems using MRTRIX 3.0.2 (Tournier et al., 2019). B0 images were then coregistered to the T1 images in native space using affine transformations. FA and MD images were then transformed to T1 native space using the estimated coregistration parameters and subsequently transformed to MNI space using the previously estimated non-linear transformations from DARTEL. Finally, mean FA and MD values for the genu and the corpus of the corpus callosum were calculated using the corresponding ROIs from the JHU ICBM white matter label atlas. Processing of diffusion weighted images failed for one of the 166 subjects that had received DWI and was thus excluded.

### Functional imaging data acquisition, preprocessing, and statistical analyses

Functional T2*-weighted images were assessed with echo planar imaging (EPI) multiband sequences (TR = 1,000ms, TE = 29.0ms, flip angle= 56°, FOV= 210mm, voxel size = 2.5mm isotropic, multiband acceleration factor = 4; 60 slices per run). The HRI task consisted of three runs of equal length.

Data were preprocessed and statistically analyzed using SPM 12 (Wellcome Department of Cognitive Neurology) running on Matlab 2012b (The Mathworks Inc, Natick, Massachusetts, USA). Images with excessive head motion (>2.5mm) were excluded from the analyses. This applied to *n* = 16 participants, resulting in a final sample of *n* = 139 participants. The first five functional images of each run were discarded to account for equilibrium effects. Further, images were reoriented to the SPM T1-template. To correct for remaining movement artifacts between scans, functional images were spatially realigned to the mean image using a 6 parameter rigid body transformation. Realigned images were then co-registered to the subject's individual structural image in native space. Estimated parameters, using DARTEL, were used to normalize images into the Montreal Neurological Institute (MNI) standard space. In the last step, normalized images were smoothed with an 8mm full width at half maximum Gaussian kernel.

In first-level analyses, the data was fit using a General linear model (GLM). Events were modeled as stick functions at stimulus onset (i.e., appearance of arrow; Aron and Poldrack, 2006) and convolved with a canonical hemodynamic response function. The model entailed four regressors of interest (correct reactions for congruent go, for incongruent go, for NoGo, and for stop trials) and the instruction, fixation cross, incorrect reactions for each condition, and six motion regressors as nuisance regressors. Subsequently, three different main contrasts were computed: incongruent go > congruent go, NoGo > congruent go, and stop > congruent go.

### Laterality index

The laterality of the brain activation was calculated using the LI-toolbox, version 1.3.2 (Wilke and Lidzba, 2007) on SPM 12. The laterality index for each participant was computed per main contrast from individual statistical t-maps derived from the first-level analyses. To assure that only activation in task-relevant regions was taken into account, only those voxels were included in the LI whose activity was significantly associated with the respective task (see previous section) after FWE correction at *p* < 0.05 and with a minimum cluster size of k = 10. To this end, a binary bilateral inclusion mask was created for each respective contrast. As with all visually presented imaging tasks, also the HRI task generates a lot of activation in visual areas, which, however is irrelevant for the laterality information in the current work. To reduce the influence of this visual activation on the calculation of the laterality index, we additionally used an exclusion mask of the occipital cortex for the analyses. This exclusion mask also consisted of the midline +/− 5mm, to rule out any artificial activity common in this area (Wilke and Lidzba, 2007). The laterality index is defined as:


$$LI=\frac{\sum activation_{left}-\sum activation_{right}}{\sum activation_{left}+\sum activation_{right}}$$

**Figure 1:**
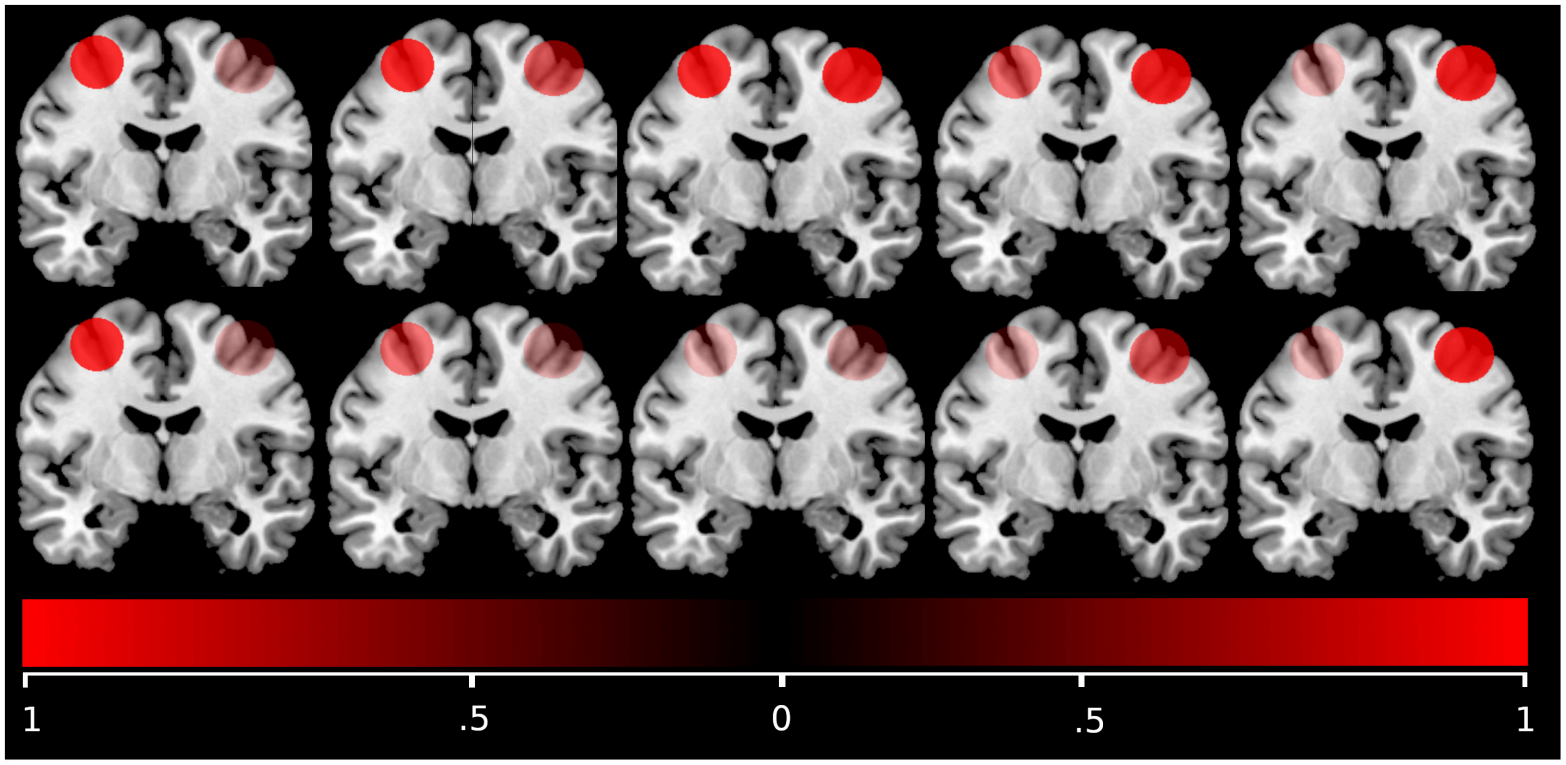
Laterality index schematic. This schematic intends to demonstrate how lateral activation scenarios are mapped by the laterality index. Very similar activation in both hemispheres corresponds to values close to zero. Higher activation in one hemisphere than in the contralateral hemisphere corresponds to values closer to one. Notably, the index is sensitive to differences in relative activation levels only and does not depend on absolute activation strength.

Here, activation refers to the first-level analyses t-maps reflecting activity during the respective tasks. Furthermore, in order to reduce the impact of outliers, a bootstrapping method was applied, iteratively drawing *n* = 100 resamples for each side from the masked and thresholded voxels on the right and left side of the input images. These resamples were tested against increasing thresholds, calculating an upper limit of 10,000 possible lateralization indices per threshold. To reduce the influence of single voxels, a lower boundary of 5 voxels per cluster surviving the threshold was defined. A trimmed mean was applied to the obtained laterality indices, only taking the mean 50% of data points into account, disregarding the upper and lower 25% of data, thereby reducing the impact of skewed distributions. To put an emphasis on voxels meaningful for the task, a so-called weighted mean (wm) was calculated from the trimmed means of all thresholds. For this, a weighting factor was applied, that is equal to the threshold survived by a given data point. Hence, laterality indices were weighted more strongly at higher thresholds (Wilke and Schmithorst, 2006). The resulting value ranges between 1 (completely left-lateralized) and –1 (completely right lateralized), with a value around 0 indicating bilaterality. As we were interested in contrasting bilateral versus unilateral brain activation, we considered the absolute of the LI throughout subsequent statistical analyses. See Figure 1 for a schematic description of the LI.

### Statistical analyses

Statistical analyses were devised in accordance with the previously published study protocol (Wolf et al., 2018). Retest effects in the training gain transfer task LPS4 were calculated descriptively from the control group as the mean difference of the assessments corresponding to the post-training and pre-training measurements with respect to short-term transfer and as the mean difference corresponding to follow-up and post-training with respect to long-term transfer. Subjects of the intervention group were then classified into short- and long-term transferers and non-transferers according to the definition given above. We calculated descriptive statistics and group comparisons for short- and long-term transferers and non-transferers for the variables age, years of education, HAWIE-R, baseline logical reasoning training task performance, and baseline LPS4 training gain transfer task performance using Wilcoxon tests. To investigate center effects, we estimated binary logistic regression models, where short-term (ST) and long-term (LT) transferer/non-transferer were set as dependent variable and study site coded as dummy variables were set as independent variables.

Subsequently, binary logistic regression analyses were conducted with transferer/non-transferer set as dependent variable, FA or MD of the genu or the corpus of the corpus callosum as independent variable and age as covariate in the subgroup of 165 subjects with DWI measures (Wolf et al., 2018). To investigate nonlinear effects, regressions were re-estimated with an added quadratic term of the independent variable. For the group of 139 subjects with fMRI data, the same regression models were estimated with the LI of the Simon, Go/NoGo and Stop tasks as independent variables. These models were also re-estimated with an added quadratic term of the respective independent variable to investigate possible non-linear effects. As subjects with high training task improvement might not have depended on more bihemispheric processing for successful training gain transfer, the models with LI as independent variable were re-estimated with cognitive training gain as additional independent variable as well as its interaction with LI. Training gains were calculated as the difference of the logical reasoning training task score between the last and the first training session. Finally, interaction effects of LIs with FA or MD values of the corpus callosum were estimated to investigate, whether the association of the structural integrity of the corpus callosum with ST and LT was dependent on more bihemispheric processing.

For the models with added quadratic or interaction terms, the difference of the Akaike Information criterion (delta AIC) (Akaike, 1998) compared to the corresponding models with quadratic or interaction terms removed was calculated. Statistical significance testing was carried out, if delta AIC was greater than two, indicating an improvement in the model. For statistically significant results, models were re-estimated using robust generalized regression models.

Statistical analyses were calculated using R version 4.4.1 (R Core Team, 2024) and the package ggplot2 version 3.5.1 was used for figures (Wickham, 2016).

## 3 Results

The mean difference for LPS4 in the control group between initial assessment and 4 weeks later (corresponding to pre-training and post-training in the intervention group) was –0.093, as well as 1.35 between the second assessment and 3 months later (corresponding to post-training and follow-up in the intervention group). Of the 180 subjects included that received the training intervention, 74 descriptively demonstrated training gains and an improvement in the LPS4 training gain transfer task at post-training, which was higher than –0.093 in the control group. They were thus classified as ST transferers according to the ex-ante definition (see above). Of these ST transferers, 19 subjects showed an improvement up until follow-up greater than 1.35 in the control group and were thus classified as LT transferers.

Group statistics did not demonstrate differences between ST/LT transferers and non-transferers with regard to age, years of education or IQ. However, ST transferers had lower baseline cognitive training task (*p* = 0.0319) and training gain transfer task performances than non-transferers (*p* < 0.0001). Likewise, LT transferers had lower baseline cognitive training task (*p* = 0.0143) and training gain transfer task (*p* = 0.0188) performances than non-transferers (see Table 1 for group statistics). Binary logistic regression analysis did not indicate significant center effects on the ST and LT transfer variables. *P*-values were *p* = 0.341 and *p* = 0.803 for the two dummy variables encoding study site.

Binary logistic regression analyses did not demonstrate any significant prediction of group membership (transferer vs. non-transferer) for FA or MD of the genu and corpus of the corpus callosum, neither between ST transferers and ST non-transferers nor between LT transferers and LT non-transferers. Likewise, laterality indices, calculated based on the activation patterns during the Simon, Go/NoGo, and Stop tasks, did not significantly predict transfer, neither between ST transferers and ST non-transferers, nor between LT transferers and LT non-transferers (see Table 2 for an overview of estimates and corresponding *p-*values). Adding baseline performance as covariate into the analyses did not change these results.

**Table 2:** Results of binary logistic regression analyses.

	**STT**		**LTT**	
**Predictor**	Estimate	p	Estimate	p
**Genu FA**	–0.6409	0.7761	2.1302	0.6089
**Corpus FA**	1.140	0.7834	–0.8979	0.8924
**Genu MD**	1.534	0.5491	2.3389	0.5385
**Corpus MD**	0.2273	0.9479	3.9795	0.4512
**LI Simon**	–1.1630	0.2360	–1.0303	0.4905
**LI Go/NoGo**	–0.3204	0.7217	0.4639	0.7289
**LI Stop**	–0.3628	0.6832	–1.5744	0.2350

STT, short-term transfer; LTT, long-term transfer; Predictor, independent variable; Estimate, corresponding regression estimate; P, corresponding *p*-value; Genu, genu of the corpus callosum; Corpus, corpus of the corpus callosum; FA, fractional diffusion anisotropy; MD, mean diffusivity; LI, lateralization index.

Models with an added quadratic term did not show an improvement over the corresponding reduced model, neither for DTI measures of the corpus callosum nor for LIs, according to delta AIC, which ranged from –1.98 to 0.99. Likewise, models with an added interaction term for LI and training gains did not show an improvement over the corresponding reduced model according to delta AIC, which ranged from –1.99 to –0.34.

Finally, two models with an interaction term for DTI measures of the corpus callosum with LIs showed a substantial improvement over the reduced model without the respective interaction term. Predicting LT transfer, delta AIC was 8.2 for the model with an interaction term of MD of the genu with the LI of the Go/NoGo task, and 3.0 for the model with an interaction term of MD of the corpus with the LI of the Go/NoGo task. The corresponding *p*-values were 0.00333 and 0.0335 for the respective interaction terms. Interactions were such that subjects with higher MD in the genu or corpus of the corpus callosum and simultaneously more bihemispheric activation during the Go/NoGo task were more likely to be LT transferers. Robust model estimates for these interaction results were somewhat lower, the interaction of the genu MD with the LI yielding a p-value of 0.0175 and the interaction of the corpus MD with the LI yielding a p-value of 0.0803, thus reducing to a trend. For an overview, please refer to Table 3.

**Table 3:** Results of interaction analyses.

**Model term**	**Estimate**	**p**	**Robust estimate**	**p**
**Genu MD**	5.3339	0.3678	3.4954	0.5861
**LI Go/NoGo**	0.2925	0.8607	0.9725	0.6089
**Interaction**	–82.7112	0.0033^*^	–69.8578	0.0175^*^
**Corpus MD**	9.2410	0.1414	5.0293	0.4529
**LI Go/NoGo**	1.1318	0.4635	0.9881	0.5376
**Interaction**	–63.0091	0.0335^*^	–54.7633	0.0803

Long term transfer was set as dependent variable, mean diffusivity (MD) of the genu or of the corpus of the corpus callosum, laterality index (LI) during the Go/NoGo task as well as their interaction term were set as independent variables. ^*^, statistically significant at *p* < 0.05 level.

## 4 Discussion

**Figure 2:**
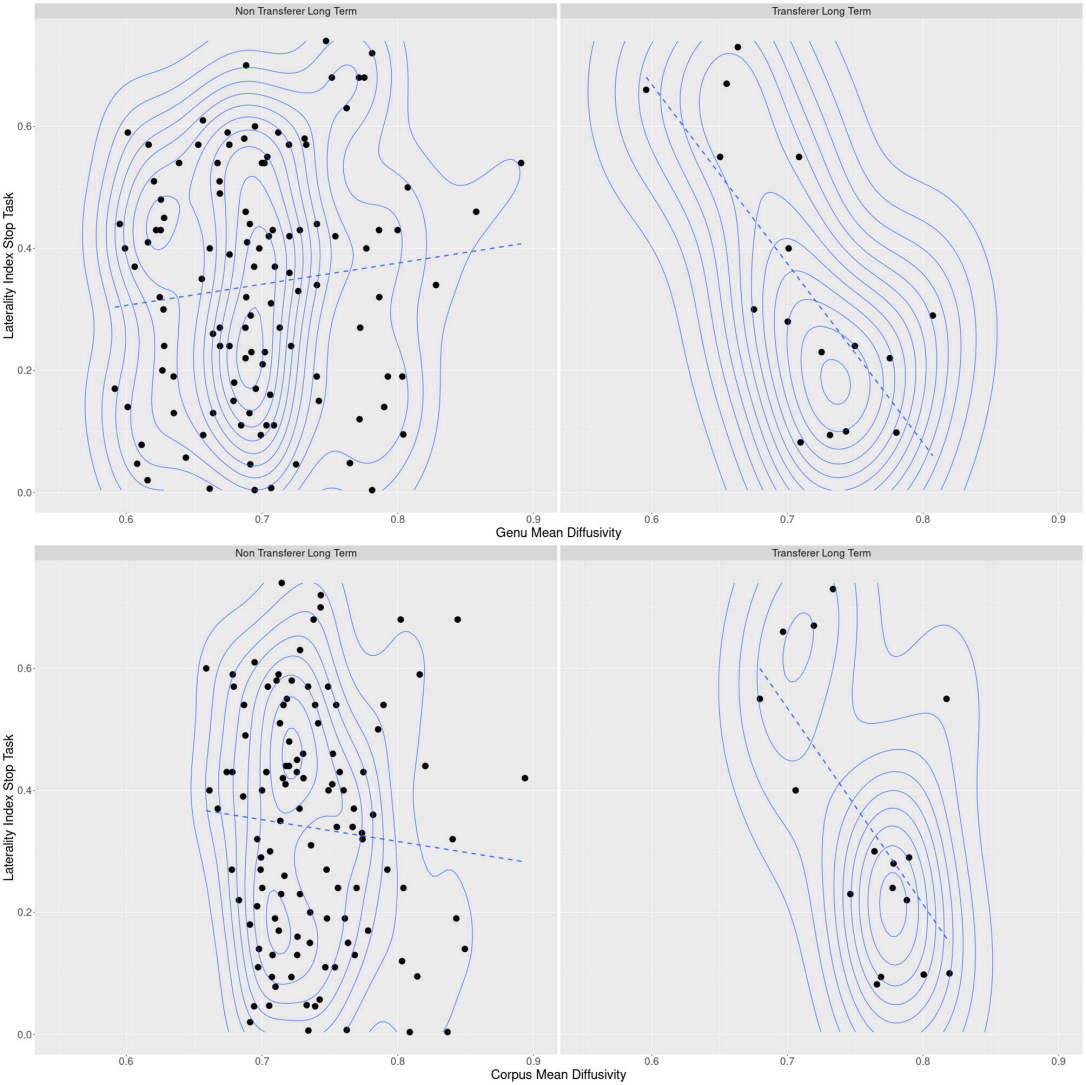
Scatter plots of long-term transferers and non-transferers of corpus callosum mean diffusivity and functional lateralization during the Stop task with superimposed density lines. The plots demonstrate interaction effects of diffusivity and lateralization from logistic regression analyses, where successful long-term transfer was more likely for subjects with both high lateralization and low diffusivity or low lateralization and high diffusivity. The dashed lines visually demonstrate a relationship between diffusivity and functional activation that is implied by their interaction effect in binary logistic regression analysis, but not statistically quantified.

Using a 4-week cognitive training intervention in a multicenter multimodal imaging study, we were able to demonstrate cognitive training gain transfer from a logical reasoning task to an untrained fluid intelligence task according to a training gain transfer measure defined ex-ante. Specifically, 74 out of 180 subjects (41.1%) showed successful short-term transfer immediately after the cognitive training intervention. The cognitive training gain transfer was stable over a period of 3 months in 19 out of these 74 subjects (25.7%), indicating long-term transfer. Short- and long-term improvements in the transfer effects were numerically greater than performance differences in the control group, indicating a true transfer effect, rather than a retest effect due to task familiarity over time. Neither successful short-term nor long-term cognitive training gain transfer were predicted by bihemispheric functional activation patterns or interhemispheric structural connectivity of the genu and corpus of the corpus callosum. However, results indicated an interaction between MD of the genu and corpus of the corpus callosum with bihemispheric functional activation during the Go/NoGo task. This interaction was such that subjects with higher levels of MD were more likely LT transferers if they showed more bihemispheric interaction, whereas subjects with lower levels of MD were more likely LT transferers if they showed less bihemispheric interaction (see Figure 2).

The proportion of ST transferers at 41.1% is lower than previously reported at 70.7% in a preceding study by our group (Wolf et al., 2014). A possible explanation might be the fairly high cognitive abilities of the sample in the study by Wolf et al. (2014), which can be inferred from the rather high IQ and educational level assessed at baseline. However, there were no differences in educational level or IQ between transferers and non-transferers in the current study. Notably though, non-transferers had a higher baseline performance in the transfer task, as well as on the training task compared to transferers. While the training task was adaptive to participants' task performance, it is conceivable that higher baseline transfer task performance left less room for potential improvement due to training gain transfer and thus led to a lower percentage of participants classified as ST transferers. However, post-training performance of ST transferers in the transfer task was higher than in ST non-transferers (*p* = 0.0018, data not shown)

While ST transfer can give insights into the effectiveness of the training, the maintenance of these training gains over time is more interesting with respect to sustained cognitive performance in aging. The proportion of participants who maintained their cognitive training gain transfer over a period of three months (i.e., LT transferers) is comparable to previous findings from our lab (Wolf et al., 2014). Comparability with other studies is impeded by the different definitions given in the literature for transfer and additionally what is categorized as near and far transfer: in the present study, we suggest to identify our short- and long-term transfer as far transfer, i.e. transfer of training gains to an untrained task from a related, yet not the same, cognitive domain. Findings of studies using this definition for transfer effects are very heterogeneous (Oberlin et al., 2022). Most meta-analyses found rather small training gains for far transfer outcomes (Nguyen et al., 2019), while a current paper even claims that far transfer effects do not exist at all (Gobet and Sala, 2023). Other training studies, using adaptive working memory training, did not find near or far transfer effects in healthy elderly participants (e.g., Guye et al., 2017; Biel et al., 2020). In contrast, a meta-analysis investigating near and far transfer of training of other cognitive domains did find evidence for far transfer effects in a few of the investigated studies (Basak et al., 2020). Furthermore, these studies commonly suggest multi-domain training with adaptive tasks, as used in the present study, to be more effective in inducing transfer effects compared to single-domain training (Nguyen et al., 2019).

Regarding the neurobiological mechanisms underlying the training gain transfer, bihemispheric integration seems to play an important role for successful LT training gain transfer in the higher level executive function task investigated in the present study. More to the point, the integrity of interhemispheric white matter connections and the corresponding degree of functional bihemispheric activation differentiates successful LT transferers from non-transferers. Subjects, whose elevated MD values in the genu and corpus of the corpus callosum indicate axonal fiber deterioration (Madden et al., 2012) in these regions are more likely to be successful LT transferers if they demonstrate more bihemispheric and functional activation. As white matter tract integrity has repeatedly been demonstrated to be impaired in healthy aging and associated with executive functioning (Sheriff et al., 2024; Piguet et al., 2009; Gunning-Dixon et al., 2009), bihemispheric functional activation could be interpreted as a form of adaption to this process that also facilitates LT transfer. In line with this view, more unilateral activation that is typical for younger persons (Cabeza, 2002; Sebastian et al., 2013a) seems to lower the probability of successful LT transfer for subjects with increased MD of the genu and corpus. Inversely, subjects with lower MD of the genu and corpus, and thus arguably less age-affected axonal fibers in these regions, were more likely to be successful LT transferers if they showed more unilateral activation as in younger subjects. One can thus speculate that in the case of more preserved interhemispheric axonal fiber connections, more bihemispheric functional activation could represent a loss of contralateral inhibitory control that impedes successful LT transfer. This dual role of bihemispheric functional activation is by a surprising analogy also manifest in monospheric stroke patients, where the degree of residual motor function determines whether contralesional hemispheric activation benefits or impedes functional recovery (Bertolucci et al., 2018).

Comparability of these findings to previous studies is limited, as to our knowledge no other studies investigated the combined influence of brain structure and function as predictors for training gain transfer. Most studies focused on brain changes induced by the training (Strenziok et al., 2014), which however, is answering a fundamentally different research question from the one investigated in the current study. However, identifying the mechanisms and modulators for successful training gain transfer is essential for the development of successful interventions to alleviate cognitive decline in healthy aging (Wolf et al., 2018). As such, the present study adds important information to the existing literature on training gain transfer and its influence on cognitive aging.

Several limitations of the current study have to be noted. First, the inclusion of only a passive control group might not have been ideal to investigate cognitive training gain transfer. Improvements from pre- to post-training are most likely due to the training in the experimental group, as they had to be larger than in the control group. However, we cannot rule out effects unassociated to the training, such as the social aspect of a training group or the structure provided by recurring appointments that might have supported a performance improvement (Gajewski et al., 2020). Future studies should exclude this by the inclusion of an active and passive control group. Moreover, statistically significant results were not corrected for family-wise error and should be regarded as tentative. Lastly, outcomes of the cognitive training on everyday functioning have not been assessed. However, it might be specifically this transition from cognitive training to everyday functioning, which is important for healthy aging. Future studies should take this into account.

## 5 Conclusion

The present study identified short- and long-term transfer effects in a group of healthy elderly participants using a 4-week cognitive training intervention. Transfer effects were maintained over time in a quarter of these participants. Successful long-term transferers showed neurobiological differences from non-transferers: subjects with more preserved interhemispheric axonal fiber connections were more likely to be successful transferers if they showed more unilateral functional activation, whereas subjects with more degraded interhemispheric axonal fiber connections were more likely to be successful transferers if they showed more bihemispheric functional activation. The quality of interhemispheric integration may determine successful cognitive training gain transfer with the potential to ultimately improve more general cognitive functioning and sustained cognitive ability in the elderly.

## Data Availability

The datasets presented in this article are not readily available because we did not obtain consent from research subjects or the ethics committee to generally share the data with the public at the time when the study was approved. The data are available to researchers involved with the consortium within the scope of shared research projects. Requests to access the datasets should be directed to oliver.tuescher@uk-halle.de; flfische@uni-mainz.de.
